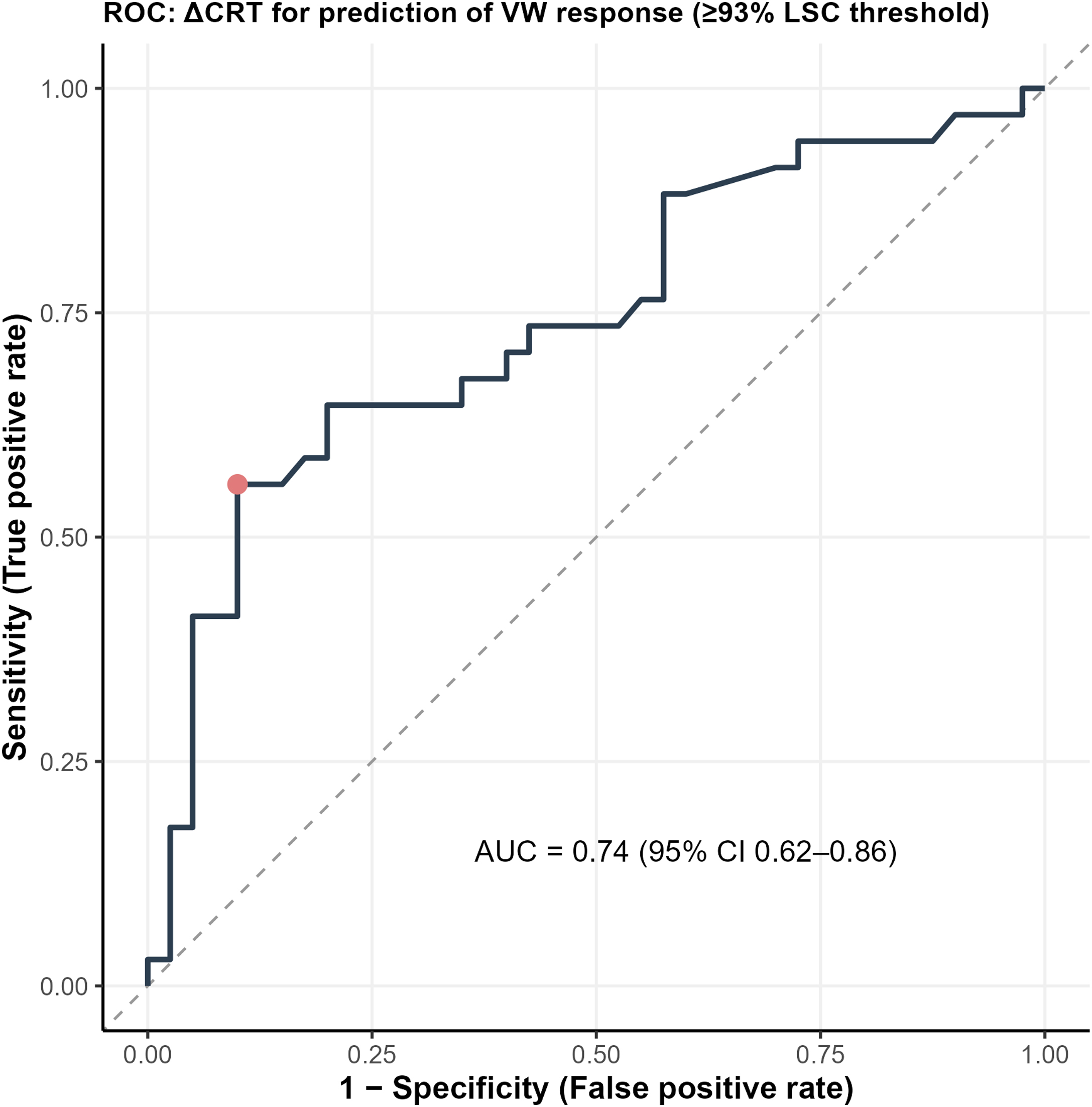# Capillary refill time changes are associated with Vascular Waterfall response in post-cardiac surgery patients

**DOI:** 10.1016/j.aicoj.2026.100105

**Published:** 2026-06-24

**Authors:** Stefan Andrei, Stéphane Bar, Maxime Nguyen, Bélaid Bouhemad, Dan Longrois, Pierre-Grégoire Guinot

**Affiliations:** a Department of Anaesthesiology and Surgical Intensive Care Unit, Groupe Hospitalier Bichat- Claude Bernard, DMU PARABOL, Assistance Publique-Hôpitaux de Paris, 75018, Paris, France; b Laboratoire de Recherche Vasculaire Translationnelle, INSERM UMR 1148, Ecole Doctorale Galilée, Université Sorbonne Paris Nord, F-75018 Paris, France; c Group of Applied Mathematics, Computational Biology and Predictive Medicine, IBENS, 75006, Paris, France; d Anaesthesiology and Critical Care Department, Amiens University Hospital, Amiens, France; e Anaesthesiology and Critical Care Department, Dijon Bourgogne University Hospital, 2 Bd Maréchal de Lattre de Tassigny, F-21000 Dijon, France; f University of Burgundy Franche Comté, LNC UMR1231, 21000 Dijon, France; g Department of Anesthesiology, Hôpital Louis Mourier, DMU PARABOL, Assistance Publique-Hôpitaux de Paris, Paris, France

**Keywords:** Capillary refill time, Vascular waterfall, Critical closing pressure, Acute circulatory failure, Microcirculation

## Abstract

**Background:**

Capillary refill time (CRT) has emerged as a validated resuscitation target in acute circulatory failure following the ANDROMEDA-SHOCK trials and the 2025 ESICM guidelines, yet the physiological mechanisms underlying its clinical utility remain incompletely understood. The vascular waterfall (VW), defined as the difference between critical closing pressure (Pcc) and mean systemic filling pressure (Pmsf), represents the pressure gradient driving microcirculatory flow. We hypothesized that CRT changes reflect VW changes during hemodynamic treatment in post-cardiac surgery patients.

**Methods:**

This was a secondary analysis of a prospective observational study conducted in post-cardiac surgery patients with acute circulatory failure (n = 74). Patients were classified into vasoplegic (n = 30), preload-dependent (n = 33), or cardiogenic (n = 11) phenotypes and treated with norepinephrine, fluid expansion, or dobutamine, respectively. Hemodynamic parameters that comprise mean arterial pressure (MAP), indexed systemic arterial resistances (SARi), indexed venous resistances (VRi) were measured before and after treatment. VW was measured using the inspiratory hold technique before and after treatment. CRT was assessed using standardized methodology. Associations between CRT and VW changes were evaluated using Spearman correlations and multivariable logistic regression.

**Results:**

Patients with prolonged baseline CRT (>3 seconds, n = 55) had significantly lower VW compared to those with normal CRT (2.8 vs 18.3 mmHg, p = 0.014). Changes in CRT correlated significantly with changes in VW across the entire cohort (ρ = −0.40, p < 0.001). Subgroup analysis showed a significant correlation in preload-dependent patients (ρ = −0.37, p = 0.032), with similar trends in vasoplegic (ρ = −0.34, p = 0.065) and cardiogenic groups (ρ = −0.20, p = 0.555). Notably, ΔCRT correlated with ΔPcc (ρ = −0.42, p < 0.001) but not with ΔPmsf (ρ = −0.03, p = 0.81), indicating that CRT changes were more closely associated with the arterial component of vascular waterfall. In multivariable logistic regression, ΔCRT independently predicted VW response (OR 0.31 per second, 95% CI 0.12−0.84, p = 0.021), alongside ΔMAP, ΔSARi, and ΔVRi. Hemodynamic phenotype did not modify this association (p = 0.90).

**Conclusions:**

CRT changes are associated with vascular waterfall changes in post-cardiac surgery patients, primarily through the relationship with critical closing pressure rather than mean systemic filling pressure. These findings may provide a physiological framework for CRT-guided resuscitation and support its potential role as a bedside marker for assessing microcirculatory perfusion pressure.

## Graphical abstract

**Figure:**
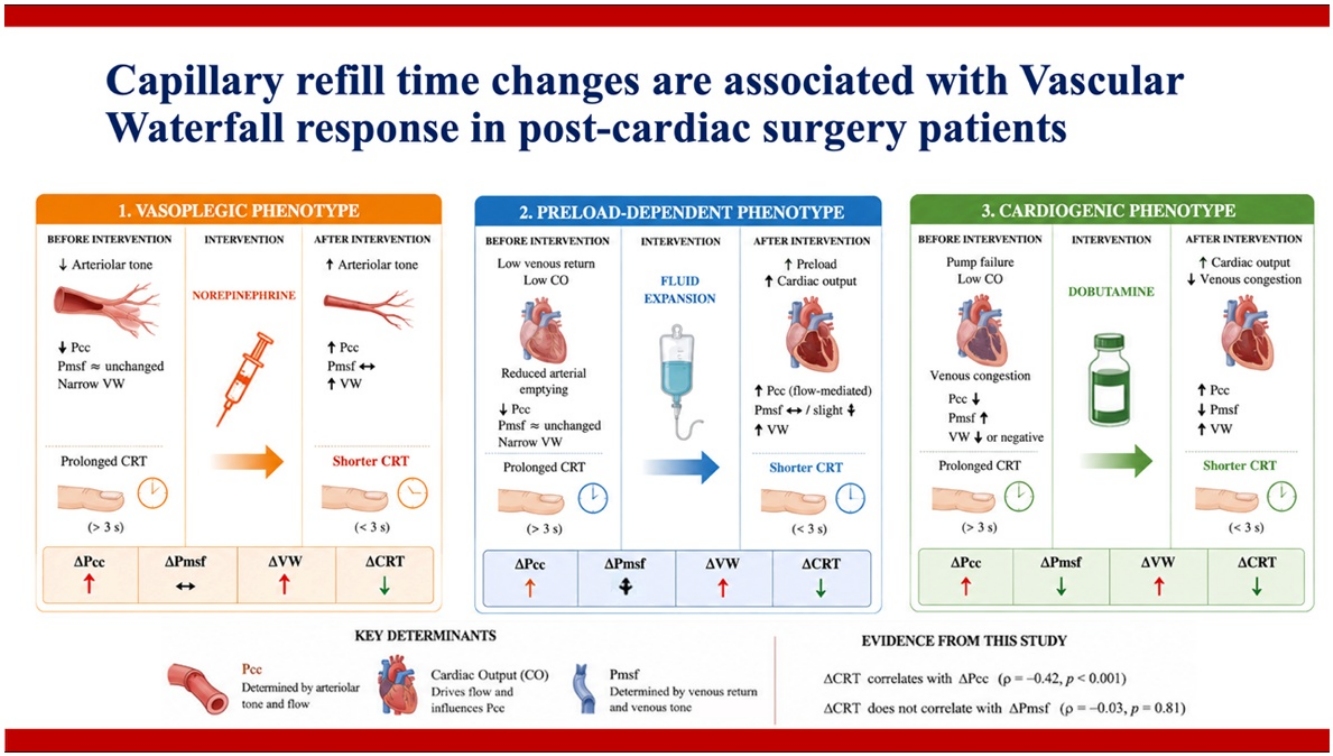

## Introduction

Acute circulatory failure is common in intensive care (ICU) patients, and after cardiac surgery [1,2]. The pathophysiology involves complex interactions between macrocirculatory dysfunction and microcirculatory impairment, ideally necessitating therapeutic strategies that address both components simultaneously [3,4]. While conventional hemodynamic targets, such as mean arterial pressure (MAP) and cardiac output (CO) guide initial resuscitation, these parameters may fail to adequately reflect tissue perfusion at the microcirculatory level [5]. During hemodynamic resuscitation, the microcirculatory response may be incoherent with changes in the macrocirculation; patients may continue to exhibit signs of tissue hypoperfusion despite the restoring of targeted MAP and CO, underscoring the need for conceptual frameworks and bedside metrics that more directly reflect the pressure gradients that drive capillary blood flow [6].

Recent evidence has reshaped hemodynamic monitoring in shock. The 2025 ESICM guidelines on circulatory shock recommend that skin perfusion should be monitored using capillary refill time (CRT) assessment, complemented by skin temperature and mottling evaluation [7]. Furthermore, the ANDROMEDA-SHOCK-2 trial demonstrated that a personalized hemodynamic resuscitation protocol targeting CRT was superior to usual care in early septic shock. Together, these establish CRT as a validated and easily available resuscitation target that can be used without complex methods. Several physiological mechanisms have been proposed to explain CRT behavior, including arteriolar smooth muscle tone, sympathetic vasoconstriction, microvascular recruitment and de-recruitment, endothelial function, and local thermoregulatory influences. While these factors are not mutually exclusive, their relative contributions to CRT in the context of acute circulatory failure remain actively debated [8,9]. This raises a fundamental question: among these candidate mechanisms, which physiological determinants link macrocirculatory interventions to CRT improvement? The vascular waterfall framework, through its arterial component (critical closing pressure), offers a testable hypothesis for one such mechanism.

The vascular waterfall (VW) concept might provide a useful physiological paradigm for understanding the dissociation between MAP targets and microcirculatory adequacy in shock [10,11]. In the systemic circulation, VW defined as the difference between critical closing pressure (Pcc) and mean systemic filling pressure (Pmsf), represents an effective downstream pressure condition shaped by arteriolar tone and critical closing behavior, rather than a direct measure of capillary perfusion pressure [12]. In post-cardiac surgery patients with vasoplegic acute circulatory failure, improving VW with vasopressors may enhance tissue perfusion markers [13]. A low or negative VW may arise from several mechanisms: reduced arteriolar tone lowering Pcc, increased venous pressure elevating Pmsf, or both. Accordingly, VW does not reflect a single hemodynamic derangement, but rather the net effective pressure head across the microcirculation, integrating arteriolar critical closing behavior on the arterial side and venous back-pressure on the downstream side. From a physiological standpoint, CRT reflects the rate of capillary blood reflow following transient mechanical occlusion and is primarily determined by upstream arterial inflow conditions. Among the components of VW, Pcc that is the pressure below which arterioles cease to conduct flow constitute the most physiologically relevant determinant of this arterial inflow. Therefore, if VW influences peripheral perfusion, CRT would be expected to track changes in Pcc more closely than Pmsf.

We hypothesized that changes in CRT reflect variations in vascular VW during hemodynamic treatment, predominantly through its arterial component (Pcc) rather than its venous component (Pmsf). Post-cardiac surgery patients represent a physiologically informative population to investigate the VW-CRT relationship, as they frequently develop acute circulatory failure with vasoplegic, preload-dependent, and cardiogenic phenotypes within a controlled environment that enables standardized hemodynamic assessment and phenotype-directed interventions. If confirmed, this relationship could provide a physiological framework for interpreting the clinical benefits observed with CRT-guided resuscitation and support the use of CRT as a bedside surrogate of VW dynamics and circulatory coherence.

Therefore, the objective of this study was to explore whether changes in CRT with hemodynamic treatment are associated with changes in VW in post-cardiac surgery patients, and to determine whether this relationship is driven by the arterial (Pcc) or venous (Pmsf) component of the vascular waterfall.

## Methods

### Study design and ethics

This was a secondary analysis of a prospective, observational, study database conducted at Amiens University Hospital, France [13]. The study protocol was approved by the local ethics committees (Comité de Protection des Personnes), and written informed consent was obtained from all patients or their legal representatives. The study is reported in accordance with STROBE guidelines for observational studies.

### Patients

Inclusion criteria were patients aged 18 years or older; arterial hypotension (mean arterial pressure <65 mmHg or systolic blood pressure <90 mmHg); presence of invasive arterial monitoring; and presence of central venous catheter. Exclusion criteria included: cardiac arrhythmias precluding accurate cardiac output measurement; severe valvular regurgitation; mechanical circulatory support; pregnancy; and refusal of consent.

Patients were managed according to standard post-cardiac surgery hemodynamic goals, with therapies adjusted based on clinical assessment and echocardiographic and invasive hemodynamic data. Hemodynamic phenotypes were defined according to the predominant circulatory profile at the time of measurement. Preload-dependent patients were not receiving vasoactive or inotropic support and demonstrated preload dependency (respiratory preload indices and/or positive passive leg raising). The vasoplegic phenotype was defined by persistent arterial hypotension that was not corrected by fluid loading and required norepinephrine support, in the absence of a low cardiac output state; these patients did not receive inotropic agents. The cardiogenic phenotype was defined by low cardiac output (CI < 2.2 l/min/m^2^ with echocardiographic evidence of left ventricular dysfunction and elevated filling pressures, requiring inotropic support (e.g., dobutamine); importantly, these patients did not receive vasopressor therapy as part of their primary hemodynamic support [14,15]. Vasoactive and inotropic infusions were maintained stable during the hemodynamic assessment period to minimize acute pharmacological effects on measured variables.

### Vascular waterfall measurement

The vascular waterfall was quantified using the inspiratory hold technique during mechanical ventilation, as previously described [12,13]. Briefly, sequential 12-second inspiratory holds were performed at increasing positive end-expiratory pressure (PEEP) levels (5, 10, and 15 cmH₂O). For each hold, arterial pressure, central venous pressure, and cardiac output were recorded during the final 3–5 seconds of the inspiratory pause, once a stable quasi-steady-state plateau had been reached after the initial transient response. Notably, the inspiratory hold protocol was limited to a maximum PEEP of 15 cmH₂O, in contrast to higher levels (up to 35 cmH₂O) used in earlier descriptions of this technique. The relationship between CVP and cardiac output was used to extrapolate Pmsf (the pressure at zero cardiac output), while the relationship between mean arterial pressure and cardiac output was used to determine Pcc (the arterial pressure at zero cardiac output). VW was calculated as Pcc minus Pmsf. Measurements were performed at baseline (T0) and after hemodynamic stabilization following phenotype-specific treatment (T1), typically 30 min after intervention initiation.

### Capillary refill time measurement

CRT was measured using a standardized technique. Firm pressure was applied to the ventral surface of the right index finger distal phalanx for 10 seconds using the examiner's fingertip. The time required for return of normal skin color following pressure release was measured using a chronometer. Three consecutive measurements were performed, and the average value was recorded. Measurements were obtained by trained investigators blinded to VW results. CRT was considered prolonged if greater than 3 seconds [16]. CRT measurements were synchronized with VW assessments at baseline and after intervention. CRT responder status was defined as a ≥10% reduction in CRT from baseline, corresponding to the inter-observer variability of standardized bedside assessment. This cutoff was chosen pragmatically to exceed the documented inter-observer variability of bedside CRT assessment [16].

### Additional hemodynamic and perfusion parameters

Hemodynamic assessment included heart rate, systolic, diastolic, and mean arterial pressure, CVP, cardiac output, and respiratory pulse pressure variation. Transthoracic echocardiography (CX50 Ultrasound System and an S5-1 Sector Array Transducer, Philips Medical System, Suresnes, France) was performed by a board-certified physician. Left ventricular ejection fraction (LVEF) was calculated using Simpson’s method on a four-chamber view. The diameter of the left ventricular outflow tract was measured on a long-axis parasternal view at the time of patient inclusion. The aortic velocity-time integral (VTIAo) was measured with pulsed Doppler on a five-chamber apical view. Stroke volume (SV; mL) was calculated as VTIAo × Aortic area and was expressed as indexed SV (SVi) = SV/body surface area (ml.m^−2^). Cardiac index (CI) (l. min^−1^.m^−2^) was calculated as SVi × heart rate (HR). Mean echocardiographic parameters were calculated from the average over five consecutives cardiac cycles and analysed retrospectively. The reproducibility of transthoracic echocardiographic CI measurements has been shown to be comparable to that of transpulmonary thermodilution, with a similar least significant change (LSC) in the range of approximately 11–14% [17,18].

From these primary measurements, vascular resistance indices were derived. Indexed systemic arterial resistance (SARi, mmHg L^−1^ min^−1^ m^−2^) was calculated as (MAP – Pcc)/CI. The indexed total peripheral resistance (TPRi) was calculated as (MAP-CVP)/CI (mmHg L^−1^ min^−1^ m^−2^). The indexed venous resistance (VRi) was calculated as (Pmsf-CVP)/CI (mmHg L^−1^ min^−1^ m^−2^). Tissue perfusion parameters included arterial lactate, central venous oxygen saturation (ScvO_2_), and central venous-to-arterial carbon dioxide difference (Pv-aCO_2_ gap). All parameters were recorded at baseline and after therapeutic intervention.

### Statistical analyses

Continuous variables are presented as median [25%–75% interquartile range] for non-normally distributed data and mean ± standard deviation for normally distributed data, as determined by the Shapiro–Wilk test. Categorical variables are presented as counts and percentages. Comparisons between time points were performed using paired t-tests or Wilcoxon signed-rank tests as appropriate. Comparisons between groups were performed using one-way ANOVA or Kruskal–Wallis test, as appropriate. Spearman’s rank test was used for assessing correlation. All hemodynamic variables, including VW, Pcc, and Pmsf, were analyzed and plotted as raw untransformed values.

The primary analysis examined the correlation between changes in CRT: ΔCRT = CRT (after therapeutic intervention) - CRT (baseline), and changes in VW: ΔVW = VW (after therapeutic intervention) - VW (baseline) using Spearman's rank correlation coefficient. Secondary analyses assessed correlations between CRT changes and changes in individual VW components (ΔPcc and ΔPmsf), as well as subgroup analyses by hemodynamic phenotype. Furthermore, exploratory logistic regression was used to identify predictors of VW response, defined as an increase in vascular waterfall ≥93% from baseline [13]. The 93% threshold represent the LSC which is the smallest change that exceeds intrinsic measurement variability and can therefore be interpreted as a true biological signal rather than random fluctuation. Odds ratios (OR) with 95% confidence intervals (CI) are reported per 1-unit change in continuous variables. For univariate logistic regression analysis, we evaluated CRT-related variables (baseline CRT, CRT > 3 seconds, CRT responder status, and ΔCRT), hemodynamic changes (ΔSAP, ΔMAP, ΔDAP, ΔPP, ΔCVP, ΔHR, ΔSV, ΔCO, ΔCI, ΔPPV), and vascular resistance indices (ΔSARi, ΔVRi). VW components (ΔPcc and ΔPmsf) were excluded from multivariate modeling to avoid circularity.

Candidate variables significant at p < 0.05 in univariable analysis were considered for the multivariable model. Given the limited number of outcome events, only physiologically pertinent variables were retained; the resulting parsimonious model also showed the lowest AIC. Collinearity was assessed using variance inflation factors (VIF), with VIF > 5 considered too high. SAP, MAP, DAP showed high collinearity (VIF > 10); MAP was retained as representative.

Receiver operating characteristic (ROC) analysis was performed to assess the discriminative ability of ΔCRT for VW response in the overall cohort and within each hemodynamic phenotype. The area under the curve (AUC) and its 95% confidence interval were computed using the DeLong method. The optimal ΔCRT cutoff was derived from the Youden index, and the corresponding sensitivity and specificity were reported.

A two-sided p-value <0.05 was considered statistically significant. Statistical analyses were performed using R software version 4.1.0 (R Foundation for Statistical Computing, Vienna, Austria). Sample size was determined by the original study design; this secondary analysis was exploratory in nature.

## Results

### Patient characteristics

Seventy-four patients were enrolled and analysed. The cohort comprised 30 patients (40.5%) with vasoplegic phenotype, 33 patients (44.6%) with preload phenotype, and 11 patients (14.9%) with cardiogenic phenotype. Baseline demographic and clinical characteristics are presented in Supplementary Table S1. Hemodynamic parameters before and after phenotype-specific treatment are presented in Table 1. All three groups demonstrated improvements in MAP following intervention: vasoplegic patients increased from 59.5 [54.9; 65.2] to 78.5 [74.0; 88.9] mmHg (p < 0.001), preload patients from 71.3 [66.7; 76.0] to 80.7 [71.0; 93.3] mmHg (p < 0.001), and cardiogenic patients from 70.7 [66.0; 77.0] to 76.7 [72.5; 85.8] mmHg (p = 0.042). Cardiac output improved significantly in all groups (p < 0.001 for all). Pulse pressure variation decreased significantly in vasoplegic and preload groups, consistent with improved preload status.

**Table 1:** Comparison of hemodynamic parameters before and after treatment for each hemodynamic intervention group.

	Vasoplegic	Preload	Cardiogenic	
Variable	N = 30	N = 33	N = 11	^2^P-value
Heart rate (bpm)				
- Before treatment	81 (18)	83 (21)	101 (25)	0.345
- After treatment	82 (18)	78 (18)	98 (24)	0.036
^1^p value	0.895	0.002	0.091	
Systolic arterial pressure (mmHg)				
- Before treatment	87 (15)	103 (16)	102 (15)	<0.001
- After treatment	121 (15)	120 (23)	115 (17)	0.463
- p value	<0.001	<0.001	0.013	
Mean arterial pressure (mmHg)				
- Before treatment	61 (10)	73 (12)	72 (8)	<0.001
- After treatment	82 (12)	82 (15)	79 (9)	0.771
- p value	<0.001	<0.001	0.041	
Diastolic arterial pressure (mmHg)				
- Before treatment	48 (9)	58 (12)	57 (6)	<0.001
- After treatment	62 (12)	63 (14)	61 (8)	0.913
- p value	<0.001	<0.001	0.153	
Central venous pressure (mmHg)				
- Before treatment	7 (4)	6 (3)	11 (5)	0.009
- After treatment	9 (4)	8 (3)	10 (5)	0.226
- p value	<0.001	<0.001	0.013	
Mean systemic pressure (mmHg),				
- Before treatment	22 [17; 27]	19 [15; 24]	34 [24; 60]	0.018
- After treatment	20 [14; 26]	18 [15; 23]	29 [22; 30]	0.011
- p value	0.28	0.611	0.052	
Critical closure pressure (mmHg)				
- Before treatment	26 [15; 35]	30 [18; 58]	20 [11; 55]	0.208
- After treatment	45 [24; 63]	51 [24; 64]	51 [28; 89]	0.915
- p value	0.003	0.048	0.095	
Vascular waterfall (mmHg)				
- Before treatment	4.6 [−9; 11.5]	10.5 [−3.6; 34.4]	−8.6 [−35.5; 39.1]	0.035
- After treatment	23.4 [−1.8; 47.2]	33.7 [5.8; 47.4]	20.5 [10; 73.1]	0.944
- p value	0.001	0.024	0.11	
Cardiac Index (L min^−1^ m^−2^)				
- Before treatment	1.7 (0.5)	1.8 (0.7)	1.6 (0.6)	0.484
- After treatment	2 (0.5)	2.2 (0.7)	2.1 (0.6)	0.737
- p value	<0.001	<0.001	0.001	
SARi (mmHg L^−1^ min^−1^ m^−2^)				
- Before treatment	4.8 [3.2; 7.8]	5.3 [3.1; 7.2]	6.1 [2.3; 12.2]	0.833
- After treatment	4.8 [1.5; 8.2]	3.3 [2.2; 6.5]	3.9 [−3.5; 7.6]	0.808
- p value	0.453	0.272	0.091	
VRi (mmHg L^−1^ min^−1^ m^−2^)				
- Before treatment	6.3 [3.8; 8.5]	5.3 [3.6; 6.5]	7.6 [5.9; 9.9]	0.044
- After treatment	8.5 [5; 13.3]	8 [5.3; 11.2]	13.3 [9.7; 26.7]	0.029
- p value	<0.001	0.001	0.021	
SvO_2_ (%)				
- Before treatment	62 [54; 68]	66 [59; 72]	62 [59; 67]	0.46
- After treatment	67 [61; 74]	64 [59; 69]	67 [60; 69]	0.35
- p value	0.002	0.719	0.062	
pCO_2_ gap (mmHg)				
- Before treatment	9 [7;11]	9 [7;11]	8 [7;12]	0.828
- After treatment	8 [6;10]	9 [7;11]	8 [6;9]	0.33
- p value	0.249	0.594	0.047	
Arterial Lactates (mmol L^−1^)				
- Before treatment	1.6 (0.5)	1.8 (1.3)	1.7 (0.5)	0.846
- After treatment	1.7 (0.7)	1.7 (0.8)	1.7 (0.6)	0.992
- p value	0.848	0.199	0.918	
Capillary refill time (seconds)				
- Before treatment	4.1 (1.1)	3.6 (1.3)	3.3 (0.5)	0.035
- After treatment	3.4 (1.1)	3.2 (1.1)	2.9 (0.8)	0.549
- p value	<0.001	0.001	0.093	
VO_2_ (mLO_2_ min^−1^)				
- Before treatment	7.8 [5.9; 9.5]	8.1 [6.3; 10]	7.3 [6.8; 7.8]	0.515
- After treatment	8.3 [7.2; 10.2]	10.3 [8.3; 12.5]	8.3 [6.9; 9.5]	0.01
- p value	0.018	<0.001	0.011	

Abbreviations: bpm, beats per minute; IQR, 25%–75% interquartile range; NE, norepinephrine; SARi, indexed systemic arterial resistances; SD, standard deviation; VRi, indexed venous resistances; VW, vascular waterfall.

Data are presented as median [interquartile range], mean (standard deviation), or n (%).

For each group, before and after treatment comparisons were performed (Wilcoxon signed rank test, ^1^p-value = comparisons before and after treatment; ^2^ p - value = comparisons between the groups of treatment (ANOVA).

### Vascular Waterfall changes with treatment

Baseline VW was lowest in the cardiogenic group (−8.6 [−32.2; 28.9] mmHg), followed by the vasoplegic group (4.6 [−7.3; 11.3] mmHg), and highest in the preload group (10.5 [−3.6; 34.4] mmHg). Following hemodynamic intervention, VW increased significantly in the vasoplegic group (to 23.4 [−0.9; 46.4] mmHg, p < 0.001) and preload group (to 33.7 [7.7; 44.8] mmHg, p = 0.023), with a non-significant improvement trend toward improvement in the cardiogenic group (to 20.5 [11.2; 53.4] mmHg, p = 0.123). The Pcc increased significantly following treatment in both vasoplegic (from 25.8 mmHg to 45.5 mmHg, p = 0.002) and preload groups (from 29.7 mmHg to 51.2 mmHg, p = 0.048). In contrast, Pmsf did not change significantly in any group.

### Capillary Refill Time changes with treatment

Baseline CRT was prolonged in all groups: 4.1 [3.5; 4.7] seconds in vasoplegic, 3.3 [2.7; 4.1] seconds in preload, and 3.3 [3.1; 3.7] seconds in cardiogenic groups. Following treatment, CRT decreased significantly in the vasoplegic group (to 3.5 [2.6; 3.8] seconds, p < 0.001) and preload group (to 3.1 [2.4; 3.9] seconds, p < 0.001), with a non-significant improvement trend in the cardiogenic group (to 2.9 [2.4; 3.5] seconds, p = 0.095).

### Relationship between CRT and Vascular Waterfall

**Fig. 1:**
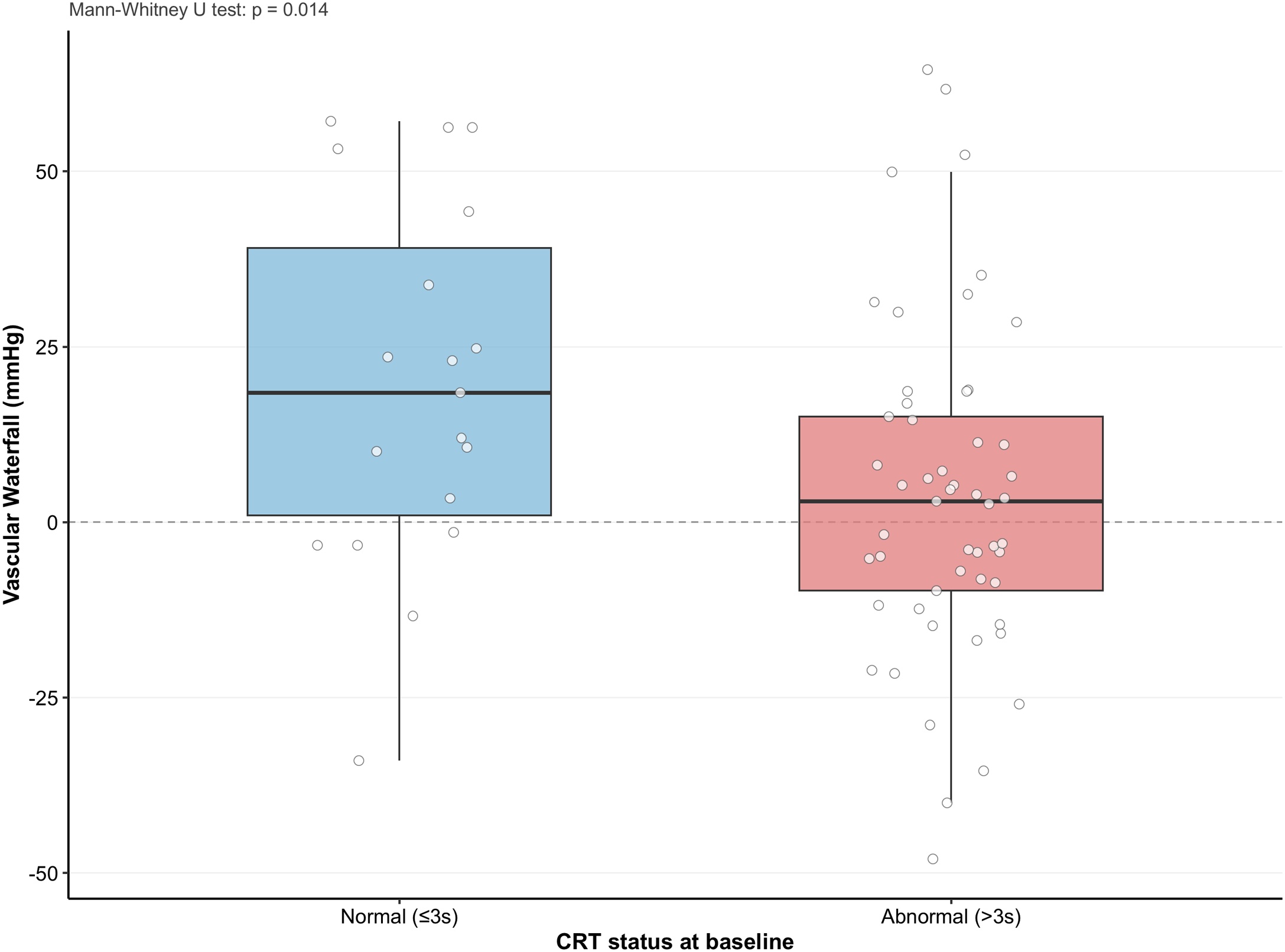
Vascular waterfall according to capillary refill time status at baseline. Patients with prolonged CRT (>3 seconds, n = 55) had significantly lower VW compared to those with normal CRT (≤3 seconds, n = 19): median 2.8 mmHg vs 18.3 mmHg, Mann-Whitney U test p = 0.014. Box plots show median (horizontal line), interquartile range (box), and 1.5 × IQR (whiskers); individual data points are overlaid as circles. VW: vascular waterfall; CRT: capillary refill time.

**Fig. 2:**
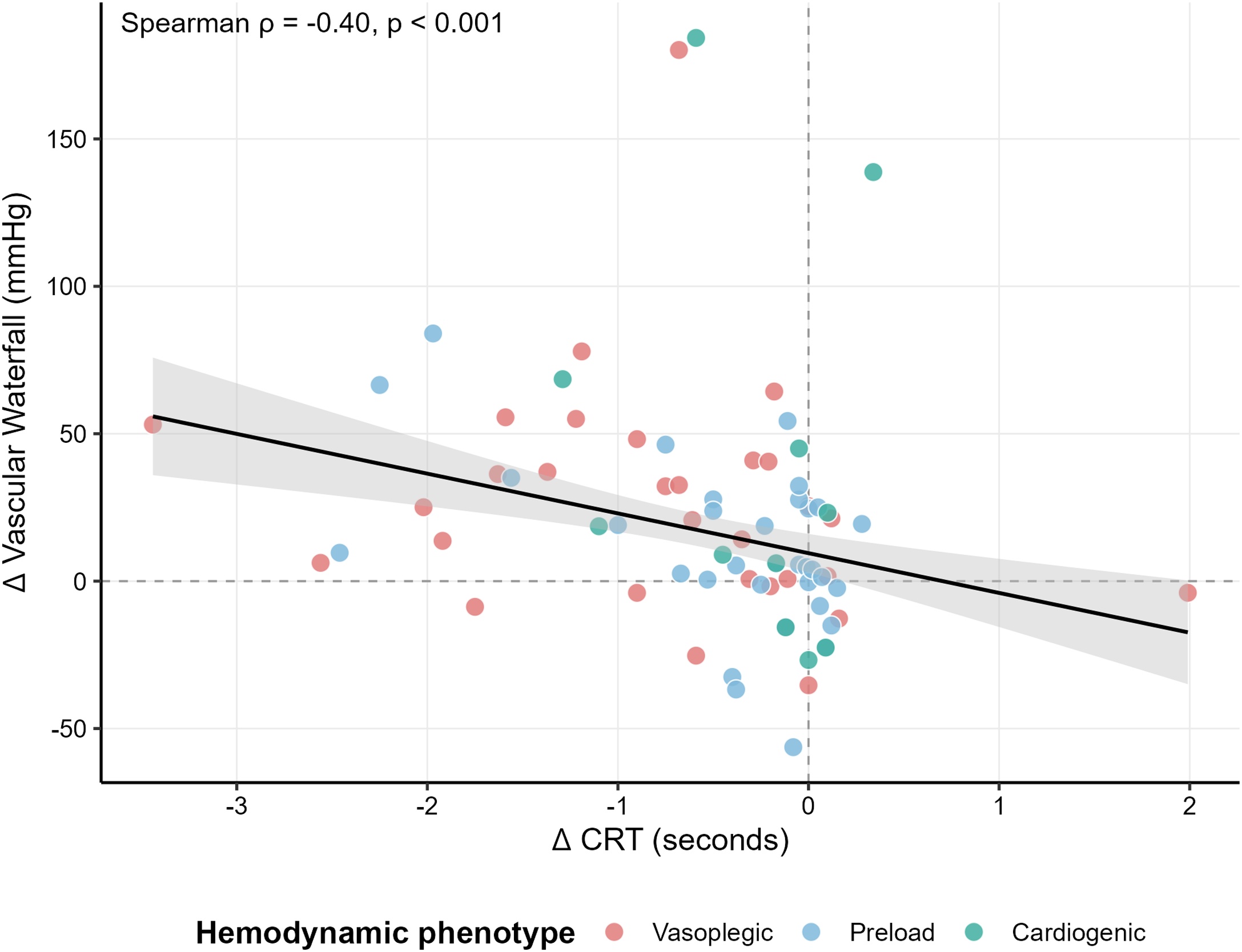
Correlation between changes in capillary refill time (ΔCRT) and changes in vascular waterfall (ΔVW) across the entire cohort. Spearman ρ = −0.40, p < 0.001. The negative correlation indicates that CRT shortening (improvement) is associated with VW expansion (improvement). Each point represents one patient and is coloured by hemodynamic phenotype (red: vasoplegic; blue: preload-dependent; green: cardiogenic). The solid black line represents a robust regression fit, with the 95% confidence interval shown as grey shading. Dashed lines indicate ΔCRT = 0 and ΔVW = 0.

**Fig. 3:**
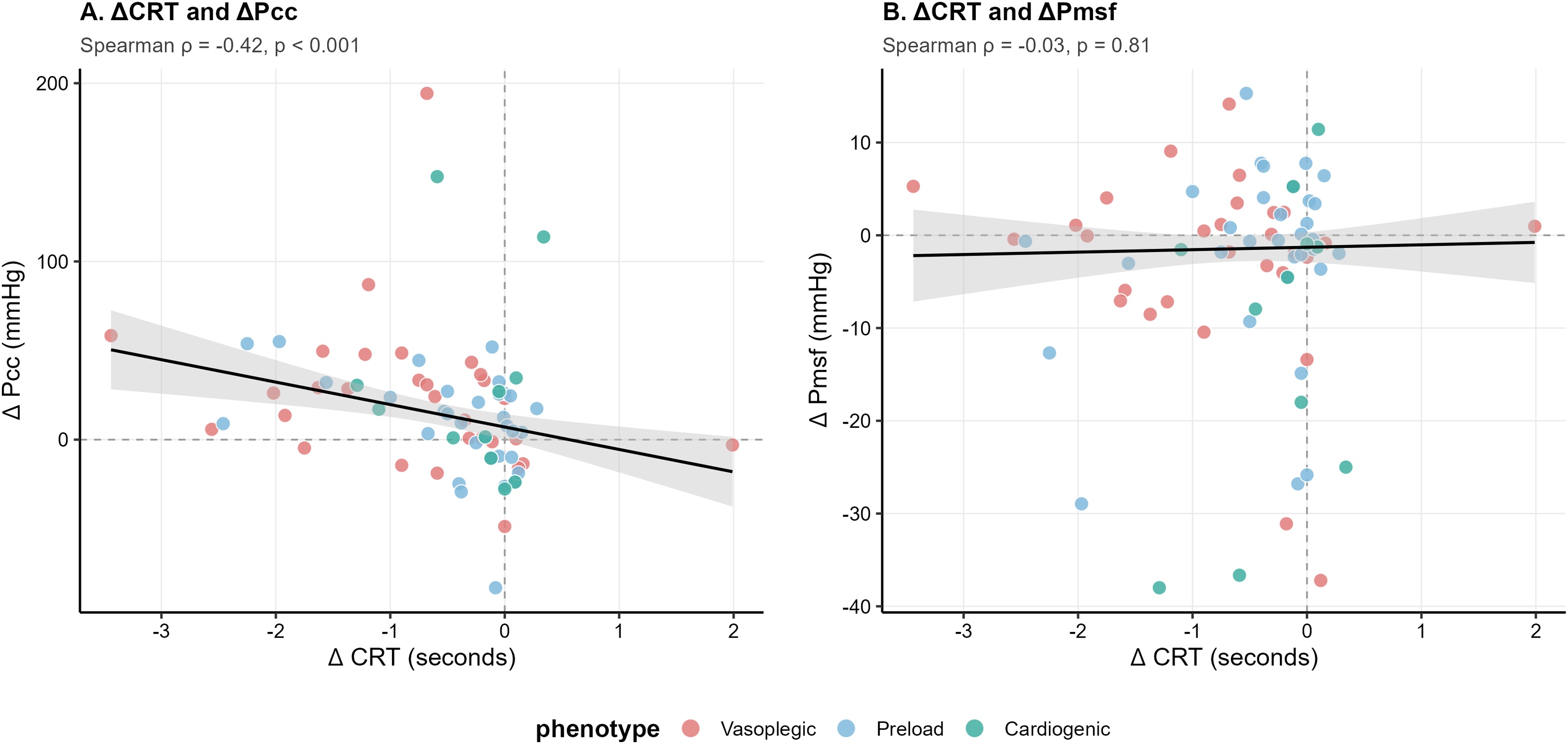
Differential correlation of ΔCRT with vascular waterfall components. A) ΔCRT correlates significantly with ΔPcc (Spearman ρ = −0.42, p < 0.001), indicating that CRT may reflect changes in the arterial component of the vascular waterfall. B) ΔCRT shows no correlation with ΔPmsf (Spearman ρ = −0.03, p = 0.81), indicating that CRT does not reflect changes in mean systemic filling pressure. Each point represents one patient and is coloured by hemodynamic phenotype (red: vasoplegic; blue: preload-dependent; green: cardiogenic). Solid lines represent robust regression fits, with 95% confidence intervals shown as grey shading. Dashed lines indicate ΔCRT = 0 and the corresponding zero on the y-axis. Pcc: critical closing pressure; Pmsf: mean systemic filling pressure.

At baseline, patients with prolonged CRT (>3 seconds, n = 55) had significantly lower VW compared to those with normal CRT (≤3 seconds, n = 19): 2.8 [−9.2; 14.7] mmHg vs 18.3 [5.4; 36.1] mmHg (p = 0.014) (Fig. 1). This baseline relationship appeared to be consistent across hemodynamic phenotypes. Changes in CRT correlated significantly with changes in VW across the entire cohort (ρ = −0.40, p < 0.001) (Fig. 2). This negative correlation indicates that decreases in CRT were associated with increases in VW. The relationship between CRT-responders and VW-responders is shown in Supplementary Table S2. When analyzing the VW components, ΔCRT correlated significantly with ΔPcc (ρ = −0.42, p < 0.001) but showed no correlation with ΔPmsf (ρ = −0.03, p = 0.81) (Fig. 3A and B).

In an exploratory ROC analysis, ΔCRT discriminated VW responders with an area under the curve (AUC) of 0.74 (95% CI 0.62−0.86); the Youden-derived optimal cutoff was - 0.67 s (sensitivity 0.56, specificity 0.90). Discriminative performance was similar across hemodynamic phenotypes, with AUCs of 0.70 (vasoplegic), 0.74 (preload-dependent), and 0.70 (cardiogenic), and Youden-optimal cutoffs ranging between −0.45 and −0.64 seconds (Supplementary Table S3, and Supplementary Fig. S3).

The final multivariate logistic regression analysis model included ΔCRT, ΔMAP, ΔSARi, and ΔVRi (Table 2). All predictors remained independently associated with VW change: ΔCRT (OR 0.31, 95% CI 0.12−0.84, P = 0.021), ΔMAP (OR 1.10, 95% CI 1.02−1.19, P = 0.021), ΔSARi (OR 0.54, 95% CI 0.39−0.76, P < 0.001), and ΔVRi (OR 0.81, 95% CI 0.69−0.96, P = 0.012). Hemodynamic phenotypes did not modify the association between ΔCRT and VW response (interaction P = 0.90) in univariate logistic regression analysis. Results were consistent across treatment groups. Variance inflation factors in the final model were all <1.2. Patients’ baseline characteristics were not associated with VW response in univariate logistic regression (Supplementary Table S4).

**Table 2:** Logistic regression analysis for VW response (defined as ΔVW ≥93%).

Variable	OR	95% CI	P value	OR	95% CI	P value
Univariate analysis				Multivariate analysis*		
*Hemodynamic parameters*						
CRT > 3 sec (baseline)	4.50	1.32−15.30	0.016			
CRT baseline, per 1 sec	1.84	1.15−2.94	0.012			
CRT responder	3.81	1.45−10.00	0.007			
ΔCRT, per 1 sec	0.28	0.13−0.64	0.002	0.31	0.12−0.84	0.021
ΔSAP, per 1 mmHg	1.03	1.00−1.06	0.039			
ΔMAP, per 1 mmHg	1.04	1.00−1.09	0.047	1.10	1.02−1.19	0.021
ΔDAP, per 1 mmHg	1.05	1.00−1.10	0.077			
ΔPP, per 1 mmHg	1.04	1.00−1.08	0.061			
ΔCVP, per 1 mmHg	0.91	0.69−1.21	0.523			
ΔHR, per 1 bpm	1.01	0.96−1.07	0.624			
ΔSV, per 1 m L	1.00	0.96−1.05	0.918			
ΔCO, per 1 L min	1.66	0.75−3.68	0.211			
ΔCardiac Index, per 1 L min^−1^ m^−2^	3.01	0.61−14.79	0.176			
ΔPPV, per 1%	0.95	0.87−1.04	0.286			
ΔSARi, per 1 mmHg L^−1^ min^−1^ m^−2^	0.65	0.53−0.81	<0.001	0.54	0.39−0.76	<0.001
ΔVRi, per 1 mmHg L^−1^ min^−1^ m^−2^	0.93	0.87−1.00	0.046	0.81	0.69−0.96	0.012
Hemodynamic phenotype			0.27			
- Vasoplegic	Ref.					
- Preload	0.44	0.16−1.20	0.109			
- Cardiogenic	0.64	0.16−2.56	0.525			

Abbreviations: OR, odds ratio; CI, confidence interval; CRT, capillary refill time; SAP, systolic arterial pressure; MAP, mean arterial pressure; DAP, diastolic arterial pressure; PP, pulse pressure; CVP, central venous pressure; HR, heart rate; SV, stroke volume; CO, cardiac output; PPV, pulse pressure variation; SARi, systemic arterial resistance index; VRi, venous resistance index, VW, vascular waterfall.

Empty cells in the multivariate columns indicate variables examined in univariable analysis but not retained in the final multivariable model.

* Final model: AIC = 57.2; pseudo-R^2^ = 0.54; VIF < 1.2 for all retained predictors.

When analyzing the correlation by phenotype, the correlation between ΔCRT and ΔVW was significant for the preload group, with a non-significant trend in the vasoplegic and cardiogenic group (Supplementary Fig. S1A–C). Per-patient trajectories of Pcc, Pmsf, and VW from baseline to post-intervention, stratified by phenotype, are shown in Supplementary Fig. S2, illustrating that Pcc increased in the majority of patients across all phenotypes, while Pmsf changes were minimal and inconsistent.

## Discussion

This study demonstrates that changes of CRT with hemodynamic treatment are associated with vascular waterfall changes. The magnitude of this correlation was moderate, indicating that CRT changes captured only part of the variability in VW changes. The current results were primarily explained through its relationship with critical closing pressure rather than mean systemic filling pressure. These new findings may provide a physiological understanding of the clinical utility of CRT-guided resuscitation, and may support its use as a bedside surrogate for microcirculatory assessment.

The main study findings provide mechanistic insight into the clinical benefits observed in the ANDROMEDA-SHOCK trials [19,20]. CRT-targeted resuscitation was associated with improved outcomes compared with lactate-guided strategies, although the physiological basis for this advantage remained uncertain. Our data suggest that targeting CRT allows clinicians to indirectly track changes in VW, and specifically in Pcc, the arterial pressure threshold governing arteriolar patency and capillary perfusion. Lactate, while a valuable marker of tissue hypoxia, is limited as a resuscitation target due to its multifactorial origin [21,22]. Consistently, a post-hoc analysis of ANDROMEDA-SHOCK showed worse outcomes in patients with normal CRT managed with a lactate-based strategy, possibly reflecting unnecessary interventions [23]. VW responders in our cohort exhibited lower baseline Pcc, higher systemic resistance, and prolonged CRT, a pattern compatible with impaired microcirculatory perfusion. This phenotype may reflect microcirculatory de-recruitment and endothelial dysfunction, but can also be explained by insufficient cardiac output, as suggested by elevated PvaCO₂ gap values. Accordingly, we do not interpret our findings as direct evidence of hemodynamic incoherence. Rather, the relationship between CRT and VW should be interpreted as a marker of Pcc-mediated coupling between arterial driving pressure and capillary perfusion, with the relative contributions of endothelial dysfunction and low-flow states remaining to be determined. This framework may help refine the physiological basis of CRT-guided resuscitation and support more targeted, physiology-driven strategies.

Another key finding of this study is the differential relationship between CRT and the two components of vascular waterfall, extending previous work on VW concept. CRT correlated with the arterial side (Pcc) but demonstrated no correlation with the venous side (Pmsf). This distinction is physiologically meaningful as the Pcc represents the minimal arterial pressure required to maintain flow through the microcirculation, essentially reflecting the pressure at which arterioles close. CRT is strongly influenced by the adequacy of peripheral arterial inflow. When Pcc is low, the pressure gradient driving capillary flow is diminished, resulting in prolonged CRT. Restoration of Pcc through vasopressor therapy re-establishes adequate arteriolar tone, increases the pressure head across the microcirculation, and accelerates capillary refill. In contrast, while Pmsf is a component of cardiac preload and venous return, it might not directly affect the arterial-to-capillary pressure gradient that determines CRT [24].

### Phenotype-specific considerations

The phenotype was not independently associated with VW response in the logistic regression analysis. Although the cohort size did not allow for more granular subgroup analyses, several physiological interpretations can be discussed with appropriate caution. Correlations were directionally consistent across phenotypes, although subgroup estimates were possibly imprecise. The correlation between CRT and VW was slightly higher in the preload-dependent subgroup, which may be consistent with the relatively straightforward hemodynamic mechanism in this phenotype. In this context, arterial hypotension secondary to reduced preload leads to compensatory vasoconstriction aimed at preserving perfusion pressure. The observed relationship may therefore primarily reflect restoration of preload and cardiac output, with subsequent increases in Pcc and VW [25], thereby restoring effective arterial flow. In this setting, the observed increase in Pcc is more likely driven by the increase in CO than by changes in SARi, which showed a numerical decrease in our cohort (even it was not significant). This may suggest that improved flow and arterial emptying, rather than a primary reduction in arterial resistance, contribute to the increase in Pcc once forward circulation is restored [26]. In contrast, Pmsf remained unchanged in our data, or exhibited only minimal variations compared with the more pronounced changes observed on the arterial side, further supporting that the main hemodynamic effects were driven by the arterial rather than venous compartment. In this framework, the observed association suggests that CRT may remain informative of microcirculatory adequacy even in preload-responsive states.

In vasoplegic shock, the primary abnormality is loss of arteriolar tone, resulting in reduced Pcc and a blunted VW [26]. Although norepinephrine restores vascular tone, increases Pcc, and may re-establish the effective pressure gradient across the microcirculation, VW did not uniformly improve in all patients [13]. This heterogeneity suggests the existence of distinct hemodynamic response profiles; however, this interpretation remains speculative in the present dataset. In this setting, improvement in CRT may reflect restoration of arteriolar tone, but this cannot be definitively inferred from the current analysis.

The cardiogenic subgroup showed a non-significant trend, likely reflecting limited statistical power. Physiologically, this phenotype is characterized by pump failure affecting both Pmsf and Pcc [25]. Inotropic support may reduce venous pressures and improve cardiac output, with secondary effects on arterial pressure and derived Pcc following restoration of flow [27]. However, these mechanisms remain hypothetical in the present study and should be interpreted as exploratory.

### Clinical relevance and future perspectives

Our findings have several clinical implications. First, capillary refill time (CRT) may serve as a rapid and readily available bedside surrogate for vascular waterfall (VW) and perfused capillary capacity in patients with acute circulatory failure. This is particularly relevant as VW assessment relies on measurement techniques that are not universally accessible. Second, persistent CRT prolongation despite restoration of macrohemodynamic parameters may reflect an incomplete tissue perfusion response and could prompt consideration of additional or alternative therapeutic strategies [9,28]. Importantly, our data do not support a meaningful modification of the CRT/VW relationship across hemodynamic phenotypes. Rather than indicating phenotype-specific mechanisms, the observed associations were consistent in direction and magnitude across groups, in line with the absence of a significant interaction. Accordingly, these findings should be interpreted as supporting a broadly conserved relationship between CRT and microcirculatory alterations, rather than distinct phenotype-dependent pathways. Further studies are warranted to validate the CRT/VW relationship in larger and more diverse setting (sepsis, surgical, medical).

### Limitations

Several limitations should be acknowledged. First, this was a secondary analysis of an existing dataset, limiting the ability to address all potential confounders. Second, the cardiogenic group was small, limiting statistical power for subgroup analysis. Third, CRT measurement is subject to inter-observer variability, although measurement technique was standardized. Fourth, the study population comprised post-cardiac surgery patients, and generalizability to other critically ill populations (e.g., medical ICU patients, septic shock) requires further validation. Fifth, cardiac output and the derived indices (cardiac index, SARi, and VRi) were measured by transthoracic echocardiography rather than a continuous reference method; although the reproducibility of echocardiographic CI is comparable to transpulmonary thermodilution, the technique is operator-dependent, and this should be considered when interpreting the resistance indices and cardiac-output–related findings. Additional factors likely influence both parameters, and the relationship should be interpreted in the context of comprehensive hemodynamic assessment rather than in isolation [8]. A substantial proportion of patients (n = 30) exhibited negative VW values at baseline; their detailed hemodynamic characteristics, compared with patients with positive baseline VW, are presented in Supplementary Table S5. A negative VW implies Pcc < Pmsf, which at first glance appears to challenge the classical Starling-resistor framework. But three non-exclusive interpretations may account for this observation. First, the inspiratory-hold technique estimates Pcc and Pmsf from a limited PEEP range (5–15 cmH₂O). In low-flow or hemodynamically unstable states, this extrapolation may be less robust and may increase variability around zero, contributing to measurement imprecision. Second, a marked reduction in arteriolar critical closing behaviour, as observed in severe vasoplegia or microcirculatory de-recruitment, may lower the effective arterial pressure floor and result in a reduced Pcc relative to Pmsf. Third, venous congestion has recently been reframed as a dynamic Starling-resistor phenomenon, in which elevated venous back-pressure may substantially influence the effective pressure gradient across the microcirculation, potentially leading to a functional “reverse waterfall” configuration in selected congestive states [29]. In our cohort, this interpretation may be particularly relevant in the cardiogenic subgroup, which exhibited the highest baseline central venous pressure and Pmsf values. Taken together, negative VW values in the present study should be interpreted cautiously, likely reflecting a combination of methodological limitations and true physiological heterogeneity, with their relative contributions still remaining to be determined. Extreme individual ΔPcc and ΔVW values observed in a small number of patients (visible as outliers in Fig. 2, Fig. 3A) likely reflect propagated uncertainty inherent to regression-based extrapolation across a limited PEEP range (5–15 cmH₂O), particularly in patients with low or unstable cardiac output. The use of Spearman's rank correlation and robust regression mitigates the influence of these values on the reported associations, and a sensitivity analysis confirmed the robustness of the primary findings.

## Conclusion

This study demonstrates that capillary refill time changes are moderately associated with vascular waterfall changes following hemodynamic treatment in post-cardiac surgery patients, specifically through its relationship with critical closing pressure. These findings may support a physiological framework for the clinical benefits observed in CRT-guided resuscitation. Further studies are needed to confirm these results in different settings.

## Authors’ contributions

Conception and study design: SA, SB, MN, BB, PGG.

Data collection: SA, SB, MN, BB, PGG.

Statistical analysis: SA, PGG.

Interpretation of data: SA, SB, MN, DL, PGG.

Writing up the first draft of the paper: SA, PGG.

Critical revision of manuscript: SB, MN, BB, DL.

Final approval of the manuscript: all authors.

## Consent for publication

All subjects received written information about the study and provided their consent to participate and publication prior to cardiac surgery.

## Ethics Approval and Consent to Participate

The study protocol was approved by the Comité de Protection des Personnes Est-I (approval number 2018-A00762-53). Written informed consent was obtained from all patients or their legal representatives.

## Declaration of Generative AI and AI-assisted technologies in the writing process

During the preparation of this work the authors used Claude version Opus 4.8 (Anthropic, San Francisco, CA, USA) and ChatGPT version 5.2 (OpenAI, San Francisco, CA, USA) for the purposes of language editing, grammar verification, and statistical code verification. After using these tools, the authors reviewed and edited the content as needed and take full responsibility for the content of the published article.

## Funding

No external funding was received. The study received no specific financial support. This study received the departmental support of CHU Dijon, France.

## Availability of Data and Materials

The datasets used and/or analysed during the current study are available from the corresponding author on reasonable request.

## Declaration of competing interest

The authors declare that the research was conducted in the absence of any commercial or financial relationships that could be construed as a potential conflict of interest.

## Figures and Tables

**Figure:**
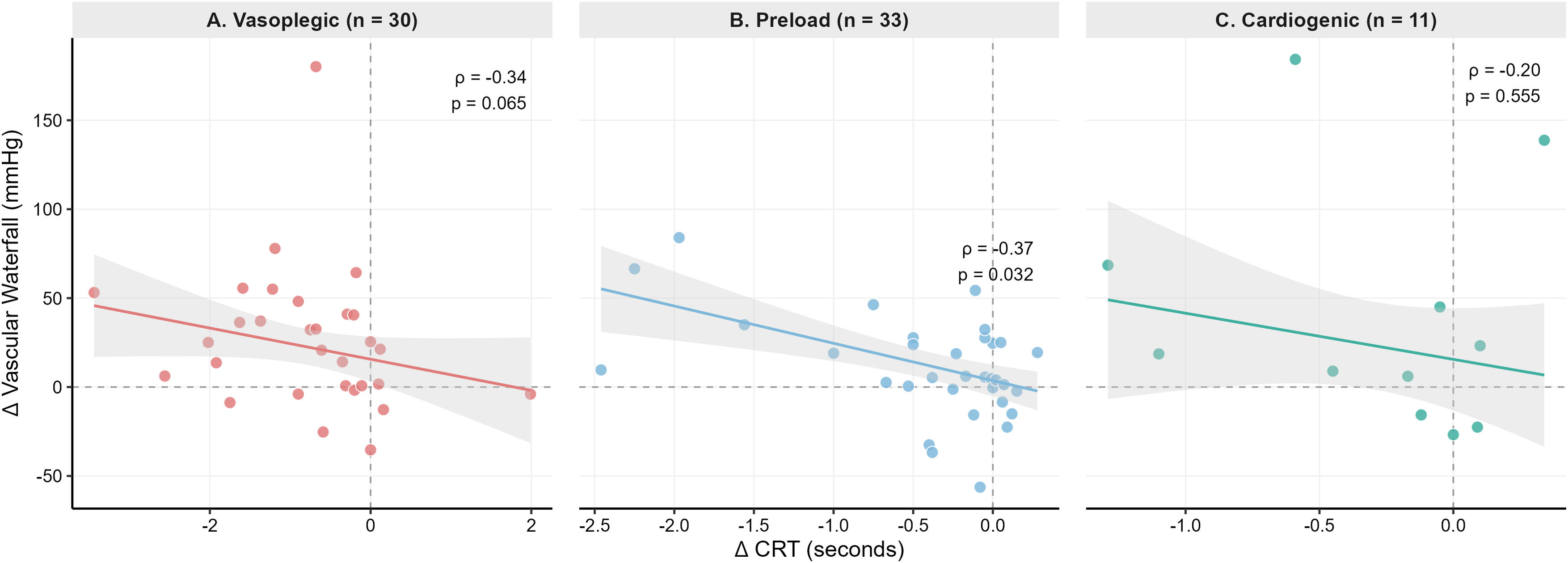


**Figure:**
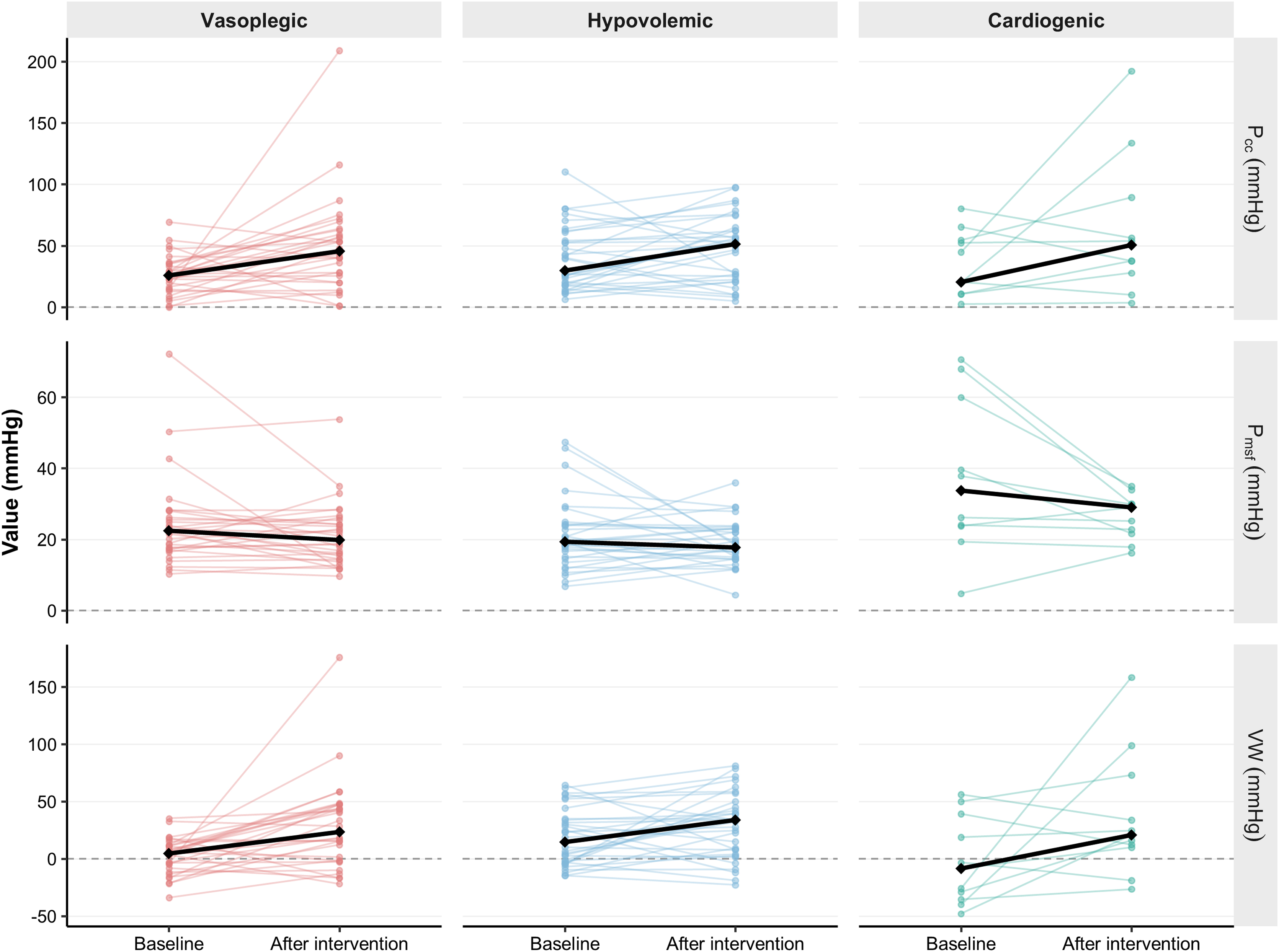


**Figure:**