# Supplementary material for: Capillary refill time changes are associated with Vascular Waterfall response in post-cardiac surgery patients
Source: Ann Intensive Care. 2026 Jun 24;16:100105. doi: 10.1016/j.aicoj.2026.100105 (PMC13330543; doi:10.1016/j.aicoj.2026.100105)
Supplement: Supplementary file 1 [file mmc1.docx]

# Supplementary Table 1. Patient characteristics

| **Variable** | **Vasoplegic**  **(n=30)** | **Preload**  **(n=33)** | **Cardiogenic (n=11)** | **P-value** |
| --- | --- | --- | --- | --- |
| Age, years | 68.5 [63.2 ;75.5] | 67.0 [58.0 ;73.0] | 64.0 [55.0 ;71.5] | 0.253 |
| Male sex, n (%) | 23 (76.7) | 27 (81.8) | 8 (72.7) | 0.799 |
| Weight, kg | 87.3 ± 19.3 | 78.4 ± 15.3 | 82.6 ± 18.9 | 0.157 |
| Body surface area, m² | 1.98 ± 0.24 | 1.88 ± 0.20 | 1.98 ± 0.24 | 0.202 |
| SAPS II score | 43 [35-51] | 38 [30-45] | 44 [38 ;52.5] | 0.239 |
| ASA class IV, n (%) | 2 (6.7) | 4 (12.1) | 6 (54.5) | 0.004 |
| Hypertension, n (%) | 23 (76.7) | 23 (69.7) | 7 (63.6) | 0.569 |
| Diabetes mellitus, n (%) | 9 (30.0) | 7 (21.2) | 2 (18.2) | 0.706 |
| Heart failure, n (%) | 12 (40.0) | 9 (27.3) | 3 (27.3) | 0.534 |
| *Outcomes* |  |  |  |  |
| ICU mortality, n (%) | 2 (6.7) | 4 (12.1) | 5 (45.5) | 0.014 |
| ICU length of stay, days | 3.0 [2.0-4.8] | 2.0 [2.0-5.0] | 7.0 [6.0-14.0] | 0.003 |

*Data are presented as median [interquartile range], mean ± standard deviation, or n (%). SAPS II: Simplified Acute Physiology Score II; ASA: American Society of Anesthesiologists; ICU: intensive care unit.*

Supplementary Table 2. Contingency table for responders to treatment.

|  | CRT non-responders | CRT-responders | Total |
| --- | --- | --- | --- |
| VW non-responders | 28 | 12 | 40 |
| VW-responders | 12 | 22 | 34 |
| Total | 40 | 34 | 74 |

*VW-responder: ≥93% increase; CRT-responder: ≥10% decrease.*

**Supplementary Table 3.** Receiver operating characteristic (ROC) analysis of ΔCRT for the prediction of vascular waterfall (VW) response in the overall cohort and stratified by hemodynamic phenotype.

| **Subgroup** | **n** | **Responders n (%)** | **AUC (95% CI)** | **Cutoff (s)** | **Sn** | **Sp** |
| --- | --- | --- | --- | --- | --- | --- |
| *Whole cohort* | 74 | 34 (46) | 0.74 (0.62-0.86) | -0.67 | 0.56 | 0.90 |
| Vasoplegic | 30 | 17 (57) | 0.70 (0.50-0.90) | -0.64 | 0.71 | 0.77 |
| Preload-dependent | 33 | 12 (36) | 0.74 (0.56-0.93) | -0.45 | 0.58 | 0.86 |
| Cardiogenic^1^ | 11 | 5 (45) | 0.70 (0.30-1.00) | -0.52 | 0.60 | 1.00 |
| *Sn: sensitivity; Sp: specificity; AUC: area under the curve; CI: confidence interval; CRT: capillary refill time; LSC: least significant change; Pcc: critical closing pressure; Pmsf: mean systemic filling pressure; VW: vascular waterfall.* | | | | | | |

*VW response was defined as a ≥93% increase in VW from baseline, corresponding to the least significant change (LSC) of the bedside measurement. Optimal cutoffs for ΔCRT (in seconds) were derived from the Youden index. Negative cutoffs indicate CRT shortening (clinical improvement). Confidence intervals for AUC were computed using the DeLong method.*

*^1^The cardiogenic subgroup should be interpreted with caution given the small sample size, as reflected in the wide confidence interval.*

**Supplemental Table 4.** Univariate logistic regression analyses for patients’ baseline characteristics and VW response (defined as ΔVW ≥93%)

| **Variable** | **OR** | **95% CI** | **P value** |
| --- | --- | --- | --- |
| Age, per 1 year | 1.00 | 0.96-1.03 | 0.800 |
| Male sex | 0.81 | 0.27-2.46 | 0.714 |
| SAPS II, per 1 point | 0.97 | 0.93-1.01 | 0.159 |
| Hypertension | 0.53 | 0.19-1.48 | 0.227 |
| Heart failure | 1.63 | 0.61-4.35 | 0.327 |
| Valvular heart disease | 1.11 | 0.44-2.76 | 0.830 |
| COPD | 0.77 | 0.12-4.91 | 0.783 |
| Smoking | 0.91 | 0.35-2.35 | 0.844 |
| Diabetes | 1.67 | 0.57-4.86 | 0.349 |
| Dyslipidemia | 1.20 | 0.48-3.02 | 0.694 |
| Chronic kidney disease | 0.27 | 0.03-2.57 | 0.256 |
| Abbreviations : OR, odds ratio; CI, confidence interval; COPD, chronic obstructive pulmonary disease; SAPS II, Simplified Acute Physiology Score II; VW, Vascular Waterfall. | | | |

**Supplementary Table 5.** Comparison of hemodynamic parameters before and after treatment for negative versus positive baseline Vascular Waterfall

| **Variable** | **Baseline VW < 0**  **N= 30** | **Baseline VW > 0**  **N =44** | **^2^P-value** |
| --- | --- | --- | --- |
| Heart rate (bpm)   - *Before treatment* - *After treatment*   *^1^p value* | 84 (23)  84 (24)  0.970 | 82 (18)  81 (18)  0.172 | 0.682  0.486 |
| Systolic arterial pressure (mmHg),   - *Before treatment* - *After treatment*   *p value* | 94 (17)  121 (15)  <0.001 | 97 (18)  120 (17)  <0.001 | 0.552  0.776 |
| Mean arterial pressure (mmHg),   - *Before treatment* - *After treatment*   *p value* | 67 (12)  82 (12)  <0.001 | 68(12)  82 (13)  <0.001 | 0.800  0.771 |
| Diastolic arterial pressure (mmHg),   - *Before treatment* - *After treatment* - *p value* | 54 (11)  63 (13)  <0.001 | 54 (11)  62 (12)  <0.001 | 0.858  0.913 |
| Central venous pressure (mmHg),   - *Before treatment* - *After treatment* - *p value* | 8 (5)  9 (4)  0.001 | 7 (3)  9 (3)  <0.001 | 0.865  0.726 |
| Mean systemic pressure (mmHg),   - *Before treatment* - *After treatment* - *p value* | 24 [19;40]  22 [16;30]  0.007 | 19 [14;24]  19 [15;23]  0.852 | 0.001  0.089 |
| Critical closure pressure (mmHg),   - *Before treatment* - *After treatment* - *p value* | 37 [13;60]  45 [24;63]  <0.001 | 40 [27;55]  53 [31;68]  0.041 | <0.001  0.093 |
| Vascular waterfall (mmHg),   - *Before treatment* - *After treatment* - *p value* | -11 [-21;-4]  14 [-9;45]  <0.001 | 19 [7;35]  34 [15 ;48]  0.024 | <0.001  0.063 |
| Cardiac Index (L min^-1^ m^-2^),   - *Before treatment* - *After treatment* - *p value* | 1.6 (0.6)  2 (0.6)  <0.001 | 1.8 (0.6)  2.1 (0.7)  <0.001 | 0.181  0.525 |
| SARi (mmHg L^-1^ min^-1^ m^-2^),   - *Before treatment* - *After treatment* - *p value* | 8.3 [5.3;12.2]  5.5 [3.3;7.9]  0.001 | 3.7 [2.4;5.8]  3 [2;5.1]  0.861 | <0.001  0.043 |
| VRi (mmHg L^-1^ min^-1^ m^-2^),   - *Before treatment* - *After treatment* - *p value* | 13.3 [8.2 ;19.1]  6.9 [5.1;9.6]  <0.001 | 6.6 [4.6;9.3]  5 [3.7;7]  0.002 | <0.001  0.009 |
| SvO_2_ (%),   - *Before treatment* - *After treatment* - *p value* | 62 [57;68]  64 [59;76]  0.054 | 66 [56;69]  66 [60;78]  0.040 | 0.636  0.586 |
| pCO_2_ gap (mmHg),   - *Before treatment* - *After treatment* - *p value* | 9 [7;11]  8 [7;12]  0.681 | 9 [7;11]  8 [6;11]  0.050 | 0.589  0.324 |
| Arterial Lactates (mmol L^-1^),   - *Before treatment* - *After treatment* - *p value* | 1.7 (0.7)  1.8 (0.8)  0.187 | 1.7 (1)  1.6 (0.6)  0.382 | 0.935  0.141 |
| Capillary refill time (seconds),   - *Before treatment* - *After treatment* - *p value* | 3.9 (1.1)  3.2 (0.8)  <0.001 | 3.7 (1.1)  3.2 (1.2)  0.002 | 0.432  0.937 |
| Abbreviations: bpm - beats per minute; IQR - 25%-75% interquartile range; NE -norepinephrine; SARi - indexed systemic arterial resistances; SD - standard deviation; VRi - indexed venous resistances; VW - vascular waterfall. | | | |

*Data are presented as median [interquartile range], mean (standard deviation), or n (%).*

*For each group, before and after treatment comparisons were performed (^1^p-value = comparisons before and after treatment; ^2^ p - value = comparisons between the groups of treatment).*
