# Supplementary material for: Capillary refill time changes are associated with Vascular Waterfall response in post-cardiac surgery patients
Source: Ann Intensive Care. 2026 Jun 24;16:100105. doi: 10.1016/j.aicoj.2026.100105 (PMC13330543; doi:10.1016/j.aicoj.2026.100105)
Supplement: Supplementary file 2 [file mmc2.docx]

**STROBE Statement – Checklist**

| **Item** | **#** | **Recommendation** | **Reported** | **Page** |
| --- | --- | --- | --- | --- |
| ***Title and abstract*** | | | | |
|  | 1 | (a) Indicate the study's design with a commonly used term in the title or the abstract | Yes – Abstract ("secondary analysis of a prospective observational study") | 1–3 |
|  |  | (b) Provide in the abstract an informative and balanced summary of what was done and what was found | Yes – Abstract | 2–3 |
| ***Introduction*** | | | | |
| **Background/rationale** | 2 | Explain the scientific background and rationale for the investigation being reported | Yes – Introduction | 4–5 |
| **Objectives** | 3 | State specific objectives, including any prespecified hypotheses | Yes – Introduction, last paragraph | 5 |
| ***Methods*** | | | | |
| **Study design** | 4 | Present key elements of study design early in the paper | Yes – Methods, Study Design and Ethics | 5 |
| **Setting** | 5 | Describe the setting, locations, and relevant dates, including periods of recruitment, exposure, follow-up, and data collection | Yes – Methods, Study Design and Ethics (Amiens University Hospital, France; CPP Est-I approval 2018-A00762-53) | 5 |
| **Participants** | 6 | (a) Give the eligibility criteria, and the sources and methods of selection of participants | Yes – Methods, Patients (inclusion/exclusion criteria; hemodynamic phenotype definitions: vasoplegic, preload-dependent, cardiogenic) | 5–6 |
| **Variables** | 7 | Clearly define all outcomes, exposures, predictors, potential confounders, and effect modifiers | Yes – Methods (Vascular Waterfall Measurement, CRT Measurement, Additional Hemodynamic and Perfusion Parameters, Statistical Analyses) | 6–8 |
| **Data sources / measurement** | 8 | For each variable of interest, give sources of data and details of methods of assessment (measurement). Describe comparability of assessment methods if there is more than one group | Yes – Methods: inspiratory-hold technique for Pcc and Pmsf extrapolation at PEEP 5/10/15 cmH₂O; standardized bedside CRT technique with three averaged measurements; transthoracic echocardiography for CI and SV (LSC 11–14%, comparable to transpulmonary thermodilution) | 6–7 |
| **Bias** | 9 | Describe any efforts to address potential sources of bias | Yes – CRT measurements obtained by trained investigators blinded to VW results (Methods, CRT Measurement); vasoactive and inotropic infusions maintained stable during the hemodynamic assessment window to minimize acute pharmacological confounding (Methods, Patients); standardized CRT protocol with three consecutive measurements averaged; Spearman rank correlation and robust regression used to mitigate the influence of extreme values; residual sources of bias discussed in Limitations | 6, 7, 14–15 |
| **Study size** | 10 | Explain how the study size was arrived at | Partially – secondary analysis of the parent prospective cohort (Andrei et al., ref 13); sample size determined by the original study design; the present analysis is described as exploratory in nature (Statistical Analyses; Limitations) | 8, 14 |
| **Quantitative variables** | 11 | Explain how quantitative variables were handled in the analyses. If applicable, describe which groupings were chosen and why | Yes – Continuous variables presented as median [IQR] or mean ± SD per Shapiro–Wilk (Statistical Analyses). Categorizations: prolonged CRT >3 s (Methods, CRT Measurement); CRT responder defined as ≥10% reduction in CRT, exceeding inter-observer variability (Methods); VW response defined as ≥93% increase from baseline, corresponding to the least significant change of the bedside Pcc–Pmsf measurement and justified as smallest change exceeding intrinsic measurement variability (Statistical Analyses); hemodynamic phenotypes defined a priori (Methods, Patients) | 6–8 |
| **Statistical methods** | 12 | (a) Describe all statistical methods, including those used to control for confounding | Yes – Spearman rank correlation; Wilcoxon signed-rank / paired t-test; Kruskal–Wallis / one-way ANOVA; univariable logistic regression screening (candidate variables significant at p<0.05 considered for the multivariable model), with the parsimonious multivariable model retaining only physiologically pertinent variables (the retained model also showing the lowest AIC); variance inflation factors (VIF >5 threshold) to assess collinearity, MAP retained among collinear arterial pressures; ΔPcc and ΔPmsf excluded from multivariable modelling to avoid circularity; ROC analysis with DeLong-method 95% CI for AUC and Youden-derived optimal cutoffs | 7–8 |
|  |  | (b) Describe any methods used to examine subgroups and interactions | Yes – phenotype-stratified Spearman correlations (Supplementary Figure 1); phenotype-stratified ROC analyses (Supplementary Table 3, Supplementary Figure 3); phenotype × ΔCRT interaction term tested in logistic regression | 7–8, 10–11 |
|  |  | (c) Explain how missing data were addressed | Yes – No multiple imputation performed; complete-case analysis; cardiogenic-subgroup ROC restricted to patients with valid CRT measurements (n=11); cardiogenic subgroup explicitly flagged for limited power | 10–11, 14 |
|  |  | (d) If applicable, describe analytical methods taking account of sampling strategy | N/A – secondary analysis of a single-centre prospective cohort | – |
|  |  | (e) Describe any sensitivity analyses | Yes – Spearman rank correlation and robust regression used as the primary approach to mitigate the influence of extreme values; a sensitivity analysis confirmed the robustness of the primary correlation (Limitations). Phenotype-stratified analyses and a comparison of patients with negative vs positive baseline VW (Supplementary Table 5) were also performed. | 7–8, 14 |
| ***Results*** | | | | |
| **Participants** | 13 | (a) Report numbers of individuals at each stage of study – e.g. numbers potentially eligible, examined for eligibility, confirmed eligible, included in the study, completing follow-up, and analysed | Yes – 74 patients enrolled and analysed: 30 vasoplegic (40.5%), 33 preload-dependent (44.6%), 11 cardiogenic (14.9%) | 10 |
|  |  | (b) Give reasons for non-participation at each stage | Partially – inclusion and exclusion criteria reported; no formal participant-flow narrative for the present secondary analysis | 5–6 |
|  |  | (c) Consider use of a flow diagram | No – not provided | – |
| **Descriptive data** | 14 | (a) Give characteristics of study participants and information on exposures and potential confounders | Yes – Table 1 (hemodynamic parameters before and after intervention, by phenotype); Supplementary Table 1 (baseline demographics and clinical characteristics) | 10, 23 |
|  |  | (b) Indicate number of participants with missing data for each variable of interest | Partially – cardiogenic CRT ROC sample size (n=11) specified; otherwise complete-case analysis with no further per-variable missingness reported | 10–11 |
| **Outcome data** | 15 | Report numbers of outcome events or summary measures | Yes – Results sections (VW changes, CRT changes, CRT–VW relationship); Tables 1 and 2 | 10–11, 23–24 |
| **Main results** | 16 | (a) Give unadjusted estimates and, if applicable, confounder-adjusted estimates and their precision. Make clear which confounders were adjusted for and why they were included | Yes – Unadjusted Spearman correlations (overall and by phenotype); univariable analyses of candidate predictors and of baseline patient characteristics (the latter in Supplementary Table 4); multivariable logistic regression with ΔCRT and the adjustment set ΔMAP, ΔSARi and ΔVRi, retained on the basis of univariable significance, physiological relevance and collinearity screening (model with the lowest AIC); ΔPcc and ΔPmsf excluded to avoid circularity; collinear arterial pressures collapsed via VIF, MAP retained | 10–11, 24 |
|  |  | (b) Report category boundaries when continuous variables were categorized | Yes – CRT >3 s; CRT responder ≥10% reduction; VW response ≥93% increase (LSC-based) | 6–8, 10 |
|  |  | (c) If relevant, consider translating estimates of relative risk into absolute risk for a meaningful time period | N/A | – |
| **Other analyses** | 17 | Report other analyses done – e.g. analyses of subgroups and interactions, and sensitivity analyses | Yes – Phenotype subgroup correlations (Supplementary Figure 1); differential correlation of ΔCRT with the VW components Pcc and Pmsf (Figure 3); phenotype × ΔCRT interaction test (p=0.90); per-patient trajectories of Pcc, Pmsf and VW by phenotype (Supplementary Figure 2); ROC analysis of ΔCRT for VW response with Youden-derived optimal cutoffs, overall and by phenotype (Supplementary Figure 3, Supplementary Table 3); univariable analyses of baseline patient characteristics (Supplementary Table 4); comparison of patients with negative vs positive baseline VW (Supplementary Table 5); robust-regression sensitivity analysis confirming the primary findings | 10–11, suppl. |
| ***Discussion*** | | | | |
| **Key results** | 18 | Summarise key results with reference to study objectives | Yes – Discussion, first paragraph | 12 |
| **Limitations** | 19 | Discuss limitations of the study, taking into account sources of potential bias or imprecision | Yes – Limitations section discusses secondary-analysis design, small cardiogenic subgroup, CRT inter-observer variability, generalizability beyond post-cardiac-surgery patients, methodological constraints of inspiratory-hold extrapolation across a limited PEEP range (5–15 cmH₂O), interpretation of negative VW values and outlier handling | 14–15 |
| **Interpretation** | 20 | Give a cautious overall interpretation of results considering objectives, limitations, multiplicity of analyses, results from similar studies, and other relevant evidence | Yes – Discussion (mechanistic framework via Pcc-mediated coupling; ANDROMEDA-SHOCK context; phenotype-specific considerations with explicit hedging; explicit acknowledgement that findings are also compatible with relative cardiac-output deficit rather than direct evidence of hemodynamic incoherence) | 12–15 |
| **Generalisability** | 21 | Discuss the generalisability (external validity) of the study results | Yes – Limitations section (post-cardiac-surgery cohort; further validation required in sepsis, medical ICU, and surgical populations) | 14–15 |
| ***Other information*** | | | | |
| **Funding** | 22 | Give the source of funding and the role of the funders for the present study and, if applicable, for the original study on which the present article is based | Yes – Declarations: no external funding for the present analysis; departmental support from CHU Dijon, France | 16–17 |
